# Explaining the Linguistic Diversity of Sahul Using Population Models

**DOI:** 10.1371/journal.pbio.1000241

**Published:** 2009-11-17

**Authors:** Ger Reesink, Ruth Singer, Michael Dunn

**Affiliations:** 1 Centre for Language Studies, Radboud University, Nijmegen, the Netherlands; 2 Research Group “Evolutionary Processes in Language and Culture,” Max Planck Institute for Psycholinguistics, Nijmegen, the Netherlands; 3 Department of Linguistics, University of Melbourne, Victoria, Australia; Massey University, New Zealand

## Abstract

The region of the ancient Sahul continent (present day Australia and New Guinea, and surrounding islands) is home to extreme linguistic diversity. Even apart from the huge Austronesian language family, which spread into the area after the breakup of the Sahul continent in the Holocene, there are hundreds of languages from many apparently unrelated families. On each of the subcontinents, the generally accepted classification recognizes one large, widespread family and a number of unrelatable smaller families. If these language families are related to each other, it is at a depth which is inaccessible to standard linguistic methods. We have inferred the history of structural characteristics of these languages under an admixture model, using a Bayesian algorithm originally developed to discover populations on the basis of recombining genetic markers. This analysis identifies 10 ancestral language populations, some of which can be identified with clearly defined phylogenetic groups. The results also show traces of early dispersals, including hints at ancient connections between Australian languages and some Papuan groups (long hypothesized, never before demonstrated). Systematic language contact effects between members of big phylogenetic groups are also detected, which can in some cases be identified with a diffusional or substrate signal. Most interestingly, however, there remains striking evidence of a phylogenetic signal, with many languages showing negligible amounts of admixture.

## Abstract 2

Areal and genealogical links between the diverse ancient languages of Australia and New Guinea are investigated using a phylogenetic clustering method adopted from population genetics.

## Author Summary

About one-fifth of all the world's languages are spoken in present day Australia, New Guinea, and the surrounding islands. This corresponds to the boundaries of the ancient continent of Sahul, which broke up due to rising sea levels about 9000 years before present. The distribution of languages in this region conveys information about its population history. The recent migration of the Austronesian speakers can be traced with precision, but the histories of the Papuan and Australian language speakers are considerably more difficult to reconstruct. The speakers of these languages are presumably descendants of the first migrations into Sahul, and their languages have been subject to many millennia of dispersal and contact. Due to the antiquity of these language families, there is insufficient lexical evidence to reconstruct their histories. Instead we use abstract structural features to infer population history, modeling language change as a result of both inheritance and horizontal diffusion. We use a Bayesian phylogenetic clustering method, originally developed for investigating genetic recombination to infer the contribution of different linguistic lineages to the current diversity of languages. The results show the underlying structure of the diversity of these languages, reflecting ancient dispersals, millennia of contact, and probable phylogenetic groups. The analysis identifies 10 ancestral language populations, some of which can be identified with previously known phylogenetic groups (language families or subgroups), and some of which have not previously been proposed.

## Introduction

“Sahul” is the name of the ancient continent which gave rise to present day Australia, New Guinea, and the surrounding islands. It was first colonized by humans around 50,000 years BP and shows extreme linguistic diversity, including the 800 Papuan and 240 Australian languages, as well as around 300 members of the Austronesian (AN) language family. The Papuan and Australian languages found there today are presumably descended from languages separated by rising sea levels at the beginning of the Holocene (∼9,000 BP) [1],[2], although connections have been obscure. This sample constitutes about 20% of all known languages.

Previous attempts to generalize about the linguistic prehistory of the area have been hampered either by lack of reproducible methods [3],[4] or inadequate data samples [5] or both. Here we present a quantitative analysis of the structural diversity of the area, based on a sample of 121 languages, and show how it can be used to infer historical relations between the present languages. The results suggest a number of new significant groupings, some reformations to earlier proposals, some first tentative links across the Torres Straits, and an overall impression of language descent and admixture during perhaps the last 20,000 years.

On each of the subcontinents the generally accepted linguistic classification recognizes one large, widespread family and a number of unrelatable smaller families: in New Guinea the Papuan languages are divided into the Trans New Guinea (TNG) family with more than 300 languages and 22 smaller non-TNG families and isolates [6]; in Australia the Pama-Nyungan (PN) family with about 180 languages, and 27 smaller non-PN families [7],[8]. AN languages spread more recently (∼4,000 BP) along the north coast of New Guinea, and diverged from ∼3,200 BP onwards from New Britain further into near and remote Oceania. [9],[10].

The linguistic situation of Sahul is thus complex, combining great time depth with (in many cases) long-term and intensive contact situations. Standard lexical methods for reconstructing language history, such as the Comparative Method [11], are not applicable, since phonological and semantic drift make it impossible to identify lexical cognate characters. Hence, phylogenetic trees are not an appropriate model of language history at this depth. We do not know that all languages descend from a common ancestor at any reasonable time scale, so we cannot start off using tree building techniques which presume that all examined taxa are related. It is therefore necessary to have a clustering method to put these languages into groups that are suggested by plausible historical scenarios and which form good hypotheses for further testing. Such a method allows for (1) a rigorous formalization of the typological approach and (2) vertical transmission and horizontal diffusion of linguistic features.

For this study we objectively assess family resemblances between groups of languages by coding each language of the sample as a set of abstract structural features, and reconstruct the history of these features using a Bayesian clustering algorithm which allows for high levels of admixture [12].

Language can throw considerable light on the population history of Sahul. First, recent migrations like the AN one can be traced with precision. Second, it can be shown that linguistic boundaries in this area can persist much longer than separated biological populations, thus retaining a signal of distinct populations after the biological signal has been obscured through interbreeding.

The traditional comparative method in historical linguistics infers language phylogeny by identifying inherited changes in the phonological systems of languages through comparison of sets of lexical cognates, words aligned by form and meaning. This method cannot apply where lexical cognates cannot be identified, and so the linguistic comparative method is not indefinitely iterable. The traditional comparative method in many instances cannot be applied to all Papuan languages, due to lack of identifiable cognates and regular sound correspondences in lexical materials [6],[13]. The lack of identifiable cognates likewise means that computational phylogenetic analyses of lexical materials, as in [10], are likewise unfeasible. The current state of the art, for example, hypothesizes the existence of the TNG family on slender correspondences in pronominal forms [6].

It has however been shown that statistical methods can overcome some of these impediments [14]–[16]. These methods use abstract structural features, rather than lexical cognates, as the basis for historical inference. Structural features necessarily have a more attenuated historical signal than lexical features, since shared structural features may originate from borrowing and convergent evolution (homoplasy) as well as from inheritance. Convergent evolution will tend to create patterns in the data which do not originate from processes of shared history, and which are thus for our purposes merely noise. Our data set is designed to minimize this problem of homoplasy. Large scale chance convergence was rendered unlikely through the use of a large number of features: while it is not improbable that any particular pair of languages are identical on a feature, the more features that are identical the more improbable this is to be the result of chance. The other possible cause of homoplasy is functional dependency—the systematic covariation of features which are functionally linked. Functional dependencies in the data set were reduced by ensuring that the structural features considered in the analysis were taken from widely distributed domains, and features with logical dependencies were excluded. Borrowing of features presented a different set of issues. Given the social demographics of the area, horizontal transmission of features must be considered part of the historical signal, rather than noise. We thus adopt a model that allows one to reconstruct population history given a current signal that encodes both phylogeny and hybridization. Rather than seeing cultures or languages as tightly integrated wholes, a population-based evolutionary model traces the degree, pattern, and processes of integration of cultural/linguistic building blocks [17]–[20]. This approach is justifiable because in principle any linguistic feature can be transferred from one speech variety to another [21].

## Materials and Methods

Following an earlier study [14],[16] that investigated possible historical scenarios for both Oceanic and Papuan languages of Island Melanesia on the basis of abstract structural features, we coded a total of 121 languages for 160 characters, 155 of which are binary, one is four-state, and four are three-state characters, using a revised questionnaire compared with the one in [14],[16]. The linguistic characters are described in the Text S1.

**Figure 1:**
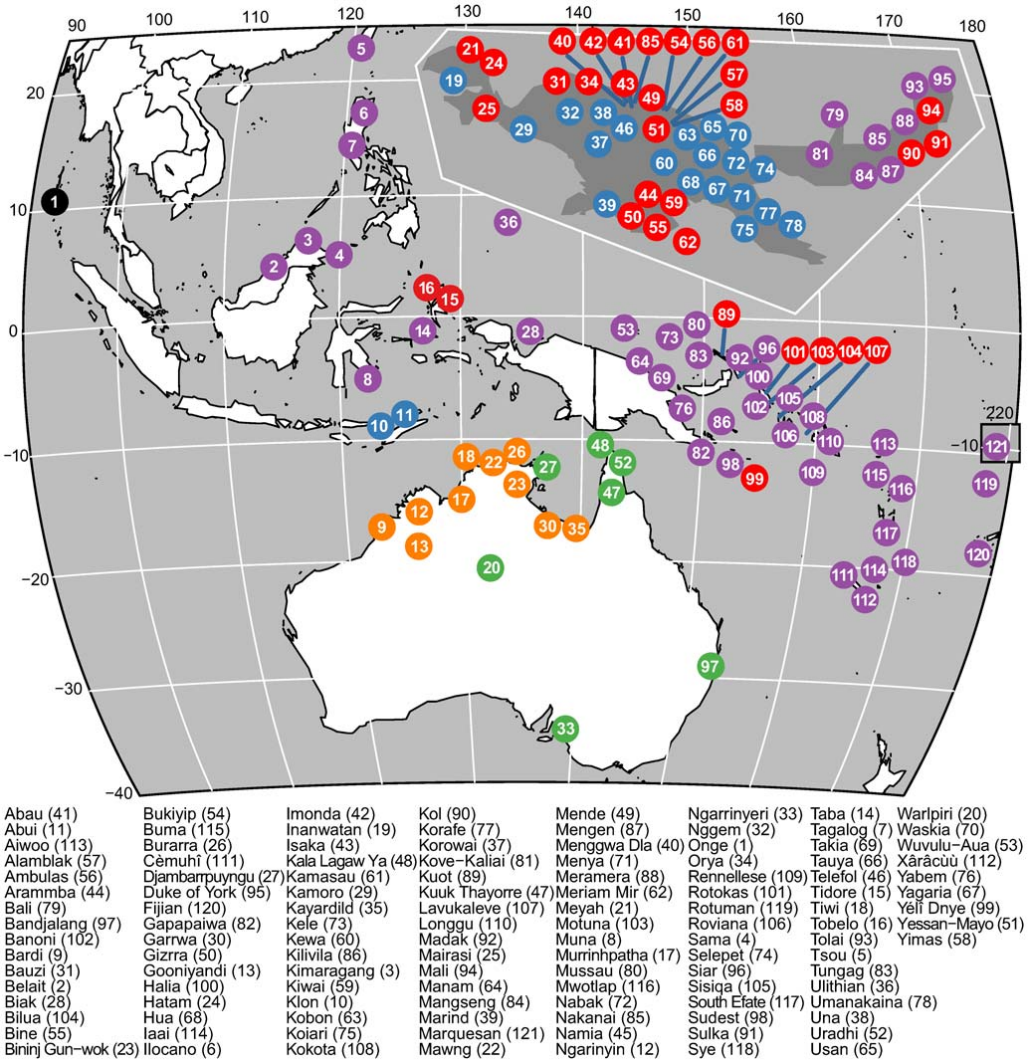
Geographic location and broad genealogical affiliation of the 121 languages in the sample (numbered west to east).

Our sample of 121 languages is made up of stratified samples of 55 Papuan languages, divided into 22 languages belonging to the putative TNG family, 33 non-TNG languages from various families; 17 Australian languages, of which seven belong to the PN family, and 10 non-PN languages belonging to various other small families; in addition, 48 AN languages, 39 of which belong to the Oceanic subgroup and 9 belong to other Western AN families; and 1 Andamese language (see map in Figure 1). The classification of the Papuan languages follows the preliminary results obtained by Ross [6] on the basis of comparison and reconstruction of pronominal paradigms. The classification of the Australian languages is based on [3] and [22]. The classification of AN languages is found in [9],[23]. The sources of language data are listed in Text S2, and the coded linguistic data are presented in Dataset S1.

The Structure algorithm [12] is a Bayesian clustering technique used to infer population structure from recombining genes (i.e., genes that are inherited from more than one parent). The method assumes a model in which there are a number (K) of unspecified or unknown populations, each of which is characterized by a set of allele frequencies at each locus. Individuals in any sample are assigned (probabilistically) to populations, or jointly to two or more populations, if their genotypes indicate that they are admixed. The method can be applied to most of the commonly used genetic markers, provided that they are not too closely linked. The Structure algorithm uses a Bayesian evolutionary model and simultaneously determines both the most likely number of ancestral linguistic groups and the most likely contribution of each of these ancestral populations to each of the observed individuals. Structure is also applicable to the analysis of language history and may prove a particularly appropriate tool when applied to typological features which can be both inherited and borrowed. The different values of the linguistic characters are the analogical equivalent of the genetic alleles, while a language is the equivalent of an individual in the biological studies. The inferred ancestral populations may be supposed to be a genealogical unit, known as a “language family,” or a group of languages whose features have converged due to an extended period of contact, known as a “Sprachbund,” or both. Analyses were carried out with the settings PLOIDY = 1 and no linkage (LINKAGE = 0). The Structure method is a character-based method which makes explicit evolutionary inferences, in contrast to distance-based clustering methods such as NeighborNet graphs (see Figure S1).

## Results

We applied Structure to the full data set of 160 structural features for 121 languages.

**Figure 2:**
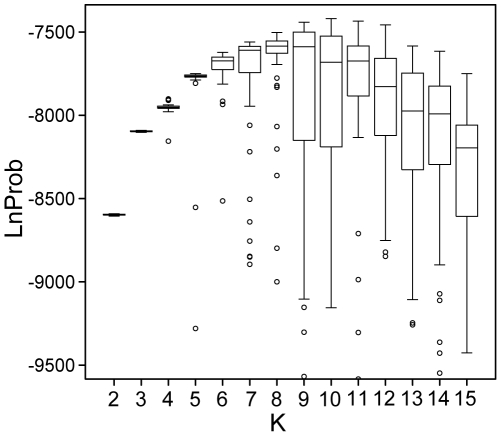
Distribution of likelihood scores for 50 independent runs of STRUCTURE at each value of K from 2 to 15.

A number of independent runs of the Structure algorithm show that different K values (the number of contributing populations) have different probabilities, steadily increasing to K10, with the higher K values having more runs with considerably lower probabilities. K values higher than 11 begin to have lower average probabilities (Figure 2).

**Figure 3:**
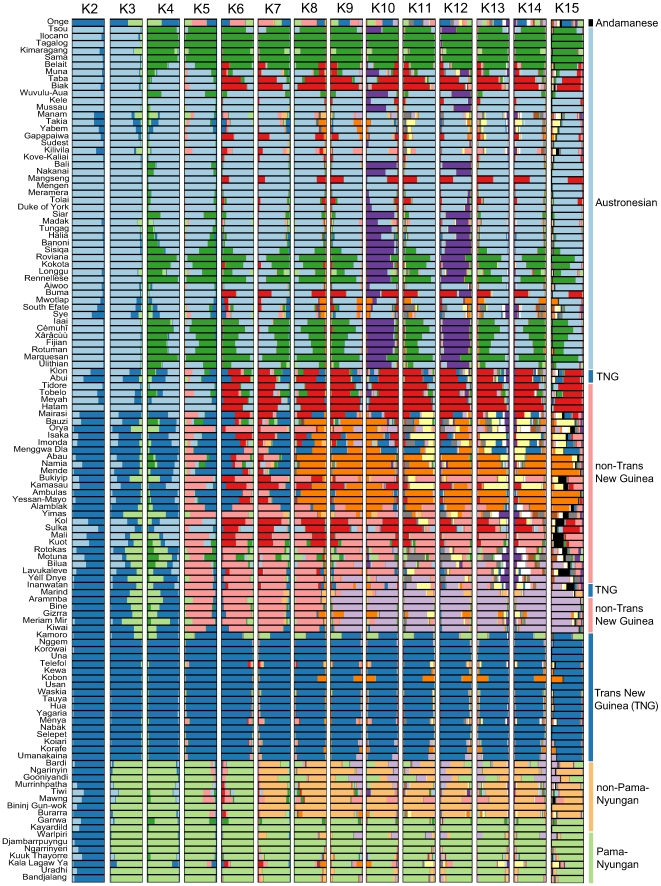
Sahul linguistic population structure. At K2 the basic contrast is between AN (Pale Blue) and all non-AN (Dark Blue), whether belonging to Papuan or Australian stocks, with some admixture in both groups. At K3 the Australian languages emerge as a solid cluster (Pale Green) within the non-AN group of K2. At K4 the AN languages are differentiated into a group which is basically the Oceanic subgroup (Pale Blue), and the remainder of western AN (Dark Green). The Oceanic languages of the Solomon Islands, New Caledonia, and Polynesia exhibit a considerable contribution from western AN as well. At K5 the Papuan languages are split into TNG (Dark Blue) and non-TNG (Pink), with some non-TNG of eastern Indonesia and New Britain showing admixture from AN clusters. At K6 a new cluster (Red) emerges, containing the Papuan languages of the Bird's Head and island Indonesia, as well as the non-TNG languages of the Bismarck archipelago. In addition, the AN languages Taba and Biak of eastern Indonesia exhibit a major contribution from this population. Some contribution is also seen in Oceanic languages east of the New Guinea mainland. At K7 we find a first diversification within the Australian set, mainly coinciding with the opposition between PN (Pale Green) and various non-PN (Yellow) families. Two non-PN languages, Garrwa and Kayardild, that had been previously classified as PN but more recently recognized as non-PN (Evans 2003: 12 [7]) cluster in our analysis with recognized PN languages. K8 exhibits a new contributing population among the non-TNG languages (Orange), present mainly in what can be identified as Northwest Papuan languages, different from other northern and southern non-TNG clusters, the latter of which is more clearly delineated in K9. At K9 the South Papuan cluster (Pale Purple) appears very strong in languages of the Trans-Fly area and Yélî Dnye of Rossel Island and has a weak contribution in Inanwatan towards the west and in Bilua of the Solomon Islands in the east. It leaves a group of languages that can be identified as Northeast Papuan. At K10 a new cluster (Dark Purple) is found among the AN languages. Signals of this population are not contiguous, suggesting two different strands in the Oceanic subgroup of the AN family: (1) a New Britain Oceanic also found in Äiwoo of the Reefs-Santa Cruz group (Pale Blue), and three languages of Vanuatu (Mwotlap, South Efate, and Sye); and (2) all other Oceanic languages (Dark Purple). Interestingly, at K11 the bifurcation of Oceanic languages of K10 disappears, while a new contributing population among the non-TNG languages can be identified. Since this K value is the first of a series with lower probabilities, we do not further discuss this, nor higher K values.

From 50 independent runs we took each K with the highest likelihood, showing that each increase in K splits one of the clusters (or founding populations) obtained with the previous value (Figure 3). The introduction of new populations thus follows the order of increasing salience; the order that new populations are detected should not be read as having any necessary relationship with chronology.

**Figure 4:**
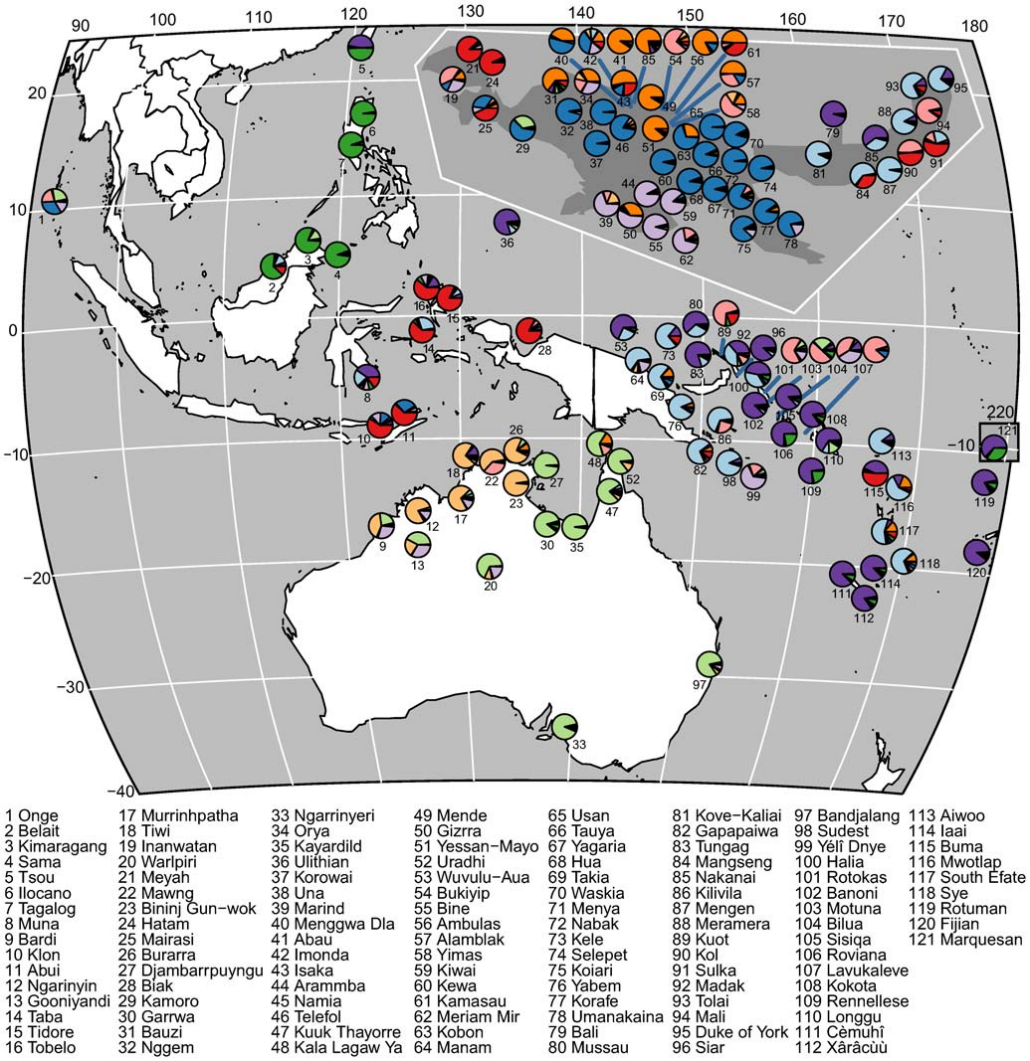
The geographic patterning of STRUCTURE results for K10. Recapitulating the K10 row from Figure 3.

The Bayes factor comparison shows that the highest likelihood K10 is 43 times more probable than K9 and 30 times more probable than K11. Figure 4 shows the results of the K10 analysis plotted onto geographic space. In the discussion of areal and phylogenetic patterns that follows, the groups are named according to their colors in Figure 3 and Figure 4.

### 1. Dark Blue

This population has a strong signal in almost all languages provisionally classified as TNG by Ross [6]. Notice that four putative TNG languages (Klon and Abui of the Timor-Alor-Pantar group, Inanwatan of the southern Bird's Head, and Marind of the south coast of Indonesian Papua) do not cluster with the rest of TNG at K6, which forms a solid block at all higher K values. Inanwatan and Marind, which may belong to a lower level family [24], do not cluster with TNG, but with other non-TNG groups. A few TNG languages show considerable admixture: Kamoro spoken along the south coast of Indonesian Papua with the Australian Light Green cluster; Kobon has some signal from Orange, and Telefol has some Pink.

Some other non-TNG languages show some contribution from the Dark Blue cluster: Mairasi of Indonesian Papua, and Imonda and Menggwa Dla, both spoken on the border.

### 2. Light Purple

This cluster contains non-TNG languages on the south coast of New Guinea, mainly the Trans-Fly region, Marind, Arammba, Bine, Gizrra, Meriam Mir, Kiwai, and also Yélî Dnye, far to the east on Rossel island. It has some contribution in Inanwatan towards the west, and in Bilua of the Solomon Islands, far to the east. A small contribution from the Light Purple cluster is also detected in Australian languages: the non-PN languages Bardi and Gooniyandi, and Warlpiri of the PN family. This is however very limited evidence for an ancient link, and the different genealogical affiliations of the Australian languages showing contributions of this cluster suggest that it may be accidental, or the product of some other factor which we have not considered.

### 3. Orange

This cluster has the strongest witnesses in Abau, Namia, Mende, Ambulas, and Yessan Mayo, all belonging to various subgroups of the non-TNG Sepik family [25]. It has also a sizeable contribution to the non-TNG languages Bauzi and Orya, I'saka, Imonda, Kamasau, and Alamblak. Some contribution is found in Inanwatan, further west, but also in widely dispersed languages: in TNG Kobon and non-TNG Gizrra and PN Kala Lagaw Ya of the Torres Strait.

### 4. Pink

In the Sepik region this cluster has the strongest witnesses in Bukiyip and Yimas, and some in Alamblak, but it characterizes mainly the so-called “East Papuan” languages of Island Melanesia, Kol, Mali, Kuot, Rotokas, Bilua, and Lavukaleve (contrary to the usual assumption, Yélî Dnye patterns with the predominantly south Papuan languages of the Light Purple cluster rather than the east Papuan languages of the Pink cluster); fainter signals of the Pink cluster are also found in Inanwatan in the west and non-PN Mawng in Australia.

### 5. Red

This cluster contains all the west Papuan languages of eastern Indonesia and the Bird's Head: Klon and Abui of the Timor-Alor-Pantar group, Tobelo and Tidore of Halmahera, and Meyah and Hatam of the Bird's Head. Mairasi, the isolate of the neck of the Bird's Head, has some contribution. A few languages along the north coast of mainland New Guinea, I'saka of the Skou family and Kamasau of the Torricelli family, also have a contribution from this cluster.

The Red is not exclusively congruent with the west Papuan languages: its signal is also present in the east Papuan languages of the Bismarck archipelago, Kol, Sulka, Mali, and Kuot, with the strongest contribution in Sulka. Moreover, there are a handful of AN languages with the same signal: Taba and Biak, belonging to the South-Halmahera-West New Guinea subgroup of the AN family. These languages are not readily distinguishable from their west Papuan neighbours on the basis of inferred population contributions. In addition, the Oceanic languages Mangseng and Tolai of New Britain, as well as Buma of the Temotu province of the Solomon Islands, each have a sizeable contribution from this cluster. We will return to this cluster in the conclusion.

The Australian languages fall into two main clusters, largely commensurate with the known division into PN and the non-PN families, mentioned in the introduction.

### 6. Light Green

This cluster largely coincides with the established PN family, in our sample represented by Warlpiri, Djambarrpuyngu, Ngarrinyeri, Kuuk-Thayorre, Kala Lagaw Ya, Uradhi, and Bandjalang. As mentioned above, Kala Lagaw Ya has some admixture from Orange, and Warlpiri from Light Purple. Garrwa and Kayardild have recently been classified [7] on the basis of comparative work as non-PN, but in our analysis they cluster firmly with PN. This agrees with earlier classifications [26],[27], which is not surprising because these were mainly based on structural features.

### 7. Yellow

Most of the non-PN languages from various families cluster as one population in our sample: Ngarinyin, Murrinhpattha, Tiwi, Mawng, Bininj Gun-Wok, and Burrara. Bardi and Gooniyandi have considerable admixture from Light Purple and Light Green as well.

As shown in Figure 3, at K10 the AN languages are divided into three clusters: Dark Green, Light Blue, and Dark Purple. Apparently this division is not very robust, as it disappears at K11, re-surfaces at K12, but is absent at all other K values.

### 8. Dark Green

This cluster contains languages of the highest nodes in the AN tree as established by the Comparative Method: Tsou from Taiwan; Ilocano, Tagalog, and Sama from the Philippines; and Sama and Belait of Borneo. Interestingly, at K10 it also shows faint signals in Oceanic languages of the Solomon Islands: Sisiqa, Longgu, Roviana, Kokota, and Renellese (a Polynesian language); of New Caledonia: Iaai, Cèmuhî, and Xârâcùù; and Polynesian languages: Fijian, Rotuman, and North Marquesan.

### 9. Light Blue

Although at K11 the AN languages are again divided into only two major clusters, there do seem to be two major strands among the Oceanic subgroup in the K value with the highest probability. One can be identified as a predominantly New Britain Oceanic cluster, which also includes Oceanic languages that have been classified as belonging to the North New Guinea linkage: Manam, Takia, and Jabêm; and the Papuan Tip family: Gapapaiwa, Sudest, and Kilivila [9].

### 10. Dark Purple

The last cluster contains all other Oceanic languages of our sample. Interestingly, Bali and Nakanai, spoken near the proposed homeland of Proto-Oceanic, belong to this cluster rather than to the Light Blue one. A number of Oceanic languages along the northern rim of New Guinea, of Island Melanesia and Vanuatu, show admixture from various Papuan clusters: Manam, Takia, and Jabêm have a contribution from Orange; Kilivila shows admixture from Pink; Mangseng, Tolai, and Buma have Red; and the Vanuatu languages Mwotlap, South Efate, and Sye have admixture from Orange.

The distribution of these linguistic populations and the degrees of their contribution to individual languages show that both phylogenetic and contact signals are detected. Millennia of contact between the small ethnolinguistic communities of New Guinea and Australia have been responsible for a great deal of convergence, making the task of establishing genealogical relationships rather difficult. We will comment on two specific instances of contact-induced convergence known in the literature, the first between Oceanic Takia and Papuan Waskia [28] and the second between three strands of Papuan: Alamblak, Yimas, and Enga [13].

After Ross had demonstrated the extent to which Oceanic Takia has been “Papuanized” on the model of a Papuan language such as neighboring Waskia, he went on to claim that the morpho-syntactic convergence of the Papuan languages of the mainland of New Guinea was due to a process by which bilingual speakers model their language on another, repeated over and over again with different language pairs [28]. Further, Foley [13], having shown the extensive diffusion of morphological patterns between Yimas, Alamblak, and Engan languages, concludes: “extended to Papuan languages generally, it is easy to see the immense problems such diffusion can create for determining the genetic affiliations and the prehistory of Papuan languages.”

However, our analysis shows these problems can be overcome. As Figure 3 shows, at all relevant K values Waskia clusters firmly with the TNG languages, while Takia clearly has a main contribution from the Oceanic cluster to which it belongs according to the Comparative Method. The contact-induced features in Takia do not immediately link it to the TNG family, but to the Orange cluster, as also found in Takia's close relatives Manam and Jabêm, all belonging to the North New Guinea linkage of the Oceanic subgroup [9]. Our methods therefore have sufficient resolution not only to unmask the imposter but also to infer the origin of its mimicked features.

At the same level of granularity, Yimas clusters with a Pink population, suggesting an affiliation with non-neighboring Bukiyip and languages of Island Melanesia, belonging to the controversial East Papuan Phylum [29]; for a critical assessment of this putative genealogical unit, see [14]–[16],[30],[31]. While Alamblak exhibits some contribution from this strand, it has a stronger affiliation with what we identify as an Orange population, comprising languages that are not immediate neighbors, nor members of the same lower-level determined families.

The Light Purple cluster, mainly found in the Trans-Fly region, cannot be explained exclusively by invoking a contact-induced process repeated over and over again that leads to massive morphosyntactic convergence. The profile of this cluster is found in languages that are geographically widely separated: Inanwatan of the Bird's Head, far to the east in Bilua of the Solomon Islands, and Yélî Dnye, far off the Papuan tip.

These examples illustrate that our set of structural features does more than just determine areal groupings blended by extensive contact, as claimed by Donohue and others [32],[33] in their critique of Dunn and colleagues [14],[15]. Structural features *can* maintain long-term signals of linguistic relatedness.

Part of the interest of the methods reported here is that when considered in tandem with independent information from the study of cognates, we are able to separate vertical from horizontal transmission in particular cases (cf. [34]). This is illustrated by the extreme convergence between AN and Papuan languages in eastern Indonesia and on New Britain (our cluster Red), and by two non-PN Australian languages, Garrwa and Kayardild, clustering with the well-established PN family in Australia.

The Structure analysis of the AN family, long known on independent cognate grounds to form one of the world's largest language families, shows that structural data clearly preserve a phylogenetic signal. By parity we may assume the same kind of phylogenetic origin for the large TNG family, where we have only fragmentary cognate confirmation. In the AN case, our analysis based on structural data clearly reflects the same internal structure of the family deduced on vocabulary grounds. The relative uniformity of the large TNG cluster perhaps makes sense in the light of the hypothesis that this family is due to an expansion dated between nine to 6,000 years ago [6],[35]. The faint admixtures across the Torres Strait could be remnants of interrelations between Papuan and Australian populations before the continents were separated about 9,000 BP.

Finally, a recent proposal in the historical linguistic literature suggests that Onge of the Andaman Islands may descend from a distant sister language of proto-AN [36]. Our results provide no support for clustering Onge with AN, or indeed any other single groupings.

## Discussion

The results of the Structure analysis demonstrate that structural features of language can be used to help clarify historical relationships. It is important however to note that these features are statistically defined clusters, and that there is not a single feature or group of features which can be taken as defining any particular linguistic genealogical unit. The typological profiles of languages, as defined by these large clusters of features, are apparently quite stable over time. It is notable also that most languages have a single cluster providing a clear majority of the contributing populations (see Figure S2).

Importantly for linguistics, this method demonstrates that computational phylogenetic methods can be applied even where processes of transmission and diffusion cannot be partitioned, and that ancient relationships can be illuminated when models of transmission and diffusion are integrated, as in the Structure method. In our study, the Structure method recapitulates known groups. Within the well established large language families (on the basis of linguistic comparative work) other details were replicated: the Oceanic subgroup was isolated within the AN family, with some areas of Oceanic showing closer affiliation with higher level members of the family; secondly, the putative TNG family appeared as a solid block (four languages tentatively included in TNG [35] were not put in this cluster); and thirdly, the Australian languages were separated from all others at K3, not showing internal differentiation until K7.

There is cause for optimism that some evidence of relationships across the Torres Straits can be found in the admixture of contributing populations (Papuan TNG and Australian PN) in Papuan Kamoro, and contributions in some Australian non-PN languages from either South-Papuan or East-Papuan populations. There are good geographic grounds for expecting such traces to be recoverable, but these results, modest as they are, are the first successes in this area.

Although we cannot specify how many different migrations have colonized Sahul since the first settlement approximately 50,000 years ago, our results indicate ancient splits into seven major plausible groups: TNG, South-Papuan, North-West Papuan, North-East Papuan, West-Papuan, PN, and non-PN. The wide-spread families (TNG and PN) on both sides of the Torres Strait divide (∼9,000 BP) are the result of more recent expansions of two of those groups, in the case of TNG probably linked to the development of agriculture, ∼9,000 to 6,000 years ago, see [35],[37].

The AN expansion is much more recent and has only had effects in eastern Indonesia, along the north coast of New Guinea and the islands east of the New Guinea mainland. We know on the basis of the comparative method correlated with archaeological data that approximately 3,200 years ago the Oceanic subgroup dispersed from its homeland on New Britain in three directions [9]: (1) back along the north coast, (2) around the eastern tip of New Guinea along the south coast, and (3) much further into the Pacific. The results of our analysis capture some of the impact of this great expansion on the languages that were already in the region. We find that in the eastern islands there are clearly distinct AN and non-AN groups, with good evidence of a deep structural phylogenetic signal, albeit with some admixture [16]. In the western islands however there is considerably more typological convergence between AN and non-AN languages (see also [38]). The linguistic population identified as Red appears to have members along the north coast (Mairasi, I'saka, and Kamasau) and on New Britain, where again both AN (Mangseng) and Papuan languages (Kol and Sulka) have contributions from the same cluster. This finding suggests an area of millennia of contact between AN and Papuan non-TNG speaking groups.

The results of the structural feature analysis do not of course replace those derived by vocabulary methods of either the traditional or the computational cladistic kinds. Where the cognate-based methods are applicable they yield finer-grained groupings than can likely be achieved by structural data alone, for the principled reason that there is a restricted design space for structural features [19]. But because known families are by-and-large recapitulated by clustering of structural features, it is reasonable to assume that hitherto unrelatable clusters discovered by the algorithm are plausible candidates for genealogical relationships. If further research shows up even a small number of possible cognates, this may be taken as more than just chance similarities.

We believe that the results obtained by this method have important ramifications for population genetic studies. When the data on mtDNA, Y chromosome, and autosomal markers are compared with the linguistic populations identified on the basis of structural features, as was done for example in [39] for Island Melanesia, we can expect significant progress in our understanding of the early colonization of Sahul.

## Supporting Information

Dataset S1 **Coded language data.** The complete data set used in this study. Numerals indicate character state, “?” indicates “unknown.”
(0.04 MB TXT)

Figure S1 **NeighborNet representation of interlinguistic structural distances.** The NeighborNet graph of the Sahul language data shows some of the same high level clusters as the STRUCTURE analysis. However the flat nature of this representation essentially forces all languages into a circular arrangement. NeighborNet only shows distance relationships, whereas STRUCTURE uses character-based evolutionary modelling [40].
(0.54 MB EPS)

Figure S2 **Distribution of STRUCTURE population inferences by proportion.** Most languages have a single ancestral population which clearly predominates.
(1.15 MB TIF)

Text S1 **Linguistic characters.** The 160 abstract structural features of language used in this study.
(0.15 MB PDF)

Text S2 **Sources of language data.** The 121 languages investigated in this study along with ISO-639-3 language codes.
(0.13 MB PDF)

## Abbreviations

AN: Austronesian
PN: Pama-Nyungan
TNG: Trans New Guinea
