# Supplementary figures and images for: Explaining the Linguistic Diversity of Sahul Using Population Models
Source: PLoS Biol. 2009 Nov 17;7(11):e1000241. doi: 10.1371/journal.pbio.1000241 (PMC2770058; doi:10.1371/journal.pbio.1000241)

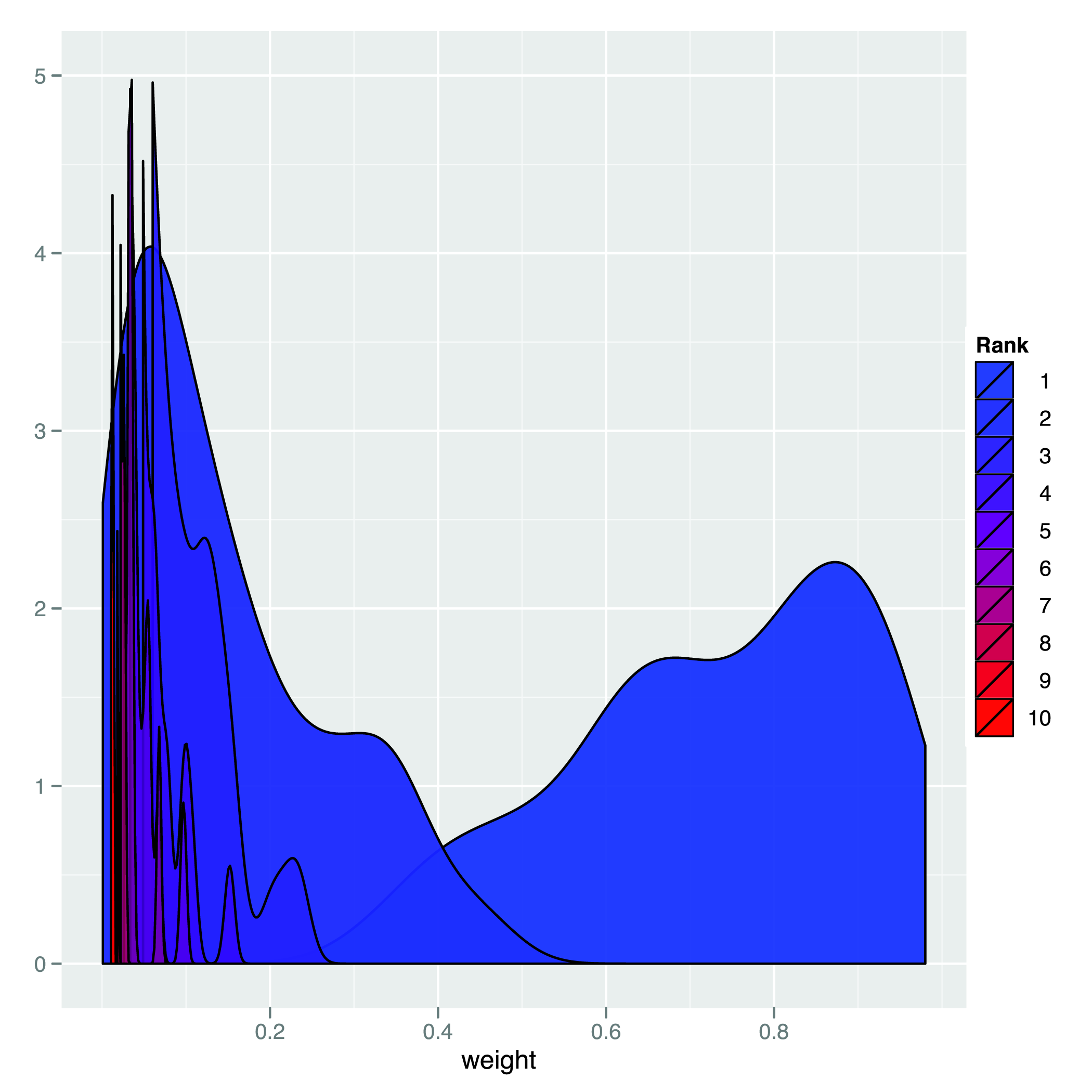

Supplement: Figure S2 — Distribution of STRUCTURE population inferences by proportion. Most languages have a single ancestral population which clearly predominates. (1.15 MB TIF) [file pbio.1000241.s003.tif]
