# Supplementary material for: Explaining the Linguistic Diversity of Sahul Using Population Models
Source: PLoS Biol. 2009 Nov 17;7(11):e1000241. doi: 10.1371/journal.pbio.1000241 (PMC2770058; doi:10.1371/journal.pbio.1000241)
Supplement: Text S1 — Linguistic characters. The 160 abstract structural features of language used in this study. (0.15 MB PDF) [file pbio.1000241.s004.pdf]

List of Linguistic characters

1. Are there as many points of articulation for nasals as there are for stops? *Only consider points of articulation where a nasal is phonetically possible* (1: present, 0: absent)

2. Is there contrast between heterorganic and homorganic sequence of nasal and velar stop? *For example, does the language permit a phonetic contrast between -nk- and -ŋk- clusters* (1: present, 0: absent)

3. Are there fricative phonemes? (1: present, 0: absent)

4. Are there phonemic prenasalised stops? (1: present, 0: absent)

5. Is there a phonemic distinction between l/r? (1: present, 0: absent)

6. Is there a phonemic velar fricative or glide? (1: present, 0: absent)

7. Is there a voicing contrast between oral (i.e. non-prenasal) stops? (1: present, 0: absent)

8. Is there a laminal/apical contrast? (1: present, 0: absent)

9. Are there retroflexed consonants? (1: present, 0: absent)

10. Is there phonemic consonant length? (1: present, 0: absent)

11. Is there phonemic vowel length? (1: present, 0: absent)

12. Are there two or more contrastive central vowels *Do not include length contrasts* (1: present, 0: absent)

13. Is there lexically determined suprasegmental prominence? *suprasegmental prominence can be loudness, duration, pitch, i.e. stress or tone phenomena (don't include phonemic vowel length)* (1: present, 0: absent)

14. Is there weight-sensitive suprasegmental prominence *suprasegmental prominence can be loudness, duration, pitch, i.e. stress or tone phenomena* (1: present, 0: absent)

15. Is there syllable position sensitive suprasegmental prominence? *suprasegmental prominence can be loudness, duration, pitch, i.e. stress or tone phenomena* (1: present, 0: absent)

16. Is there a tonal system? *I.e. two or more contrastive tones* (1: present, 0: absent)

17. Are there word-final consonants? (1: present, 0: absent)

18. Are there consonant clusters (not counting prenasalized consonants) in syllable onset? (1: present, 0: absent)

19. Are there consonant clusters (not counting prenasalized consonants) in syllable coda? (1: present, 0: absent)

20. Are there definite or specific articles? (1: present, 0: absent)

21. Is an indefinite NP obligatorily accompanied by an indefinite (or non-specific) article?

Disregard if only on personal names (1: present, 0: absent)

22. Are there prenominal articles? (1: present, 0: absent)

23. Are there postnominal articles? (1: present, 0: absent)

24. What is the relative position of numeral and noun in the NP? (*multistate* 1; Num-N; 2: N-Num; 3: both.)

25. What is the relative position of demonstrative and noun in the NP? (*multistate* 1: Dem-N; 2: N-Dem; 3: both.)

26. Are there 'discontinuous noun phrases'? *Can an argument be expressed by multiple N/NP throughout the clause > i.e. the Australian type.* (1: present, 0: absent)

27. Is there a difference between the marking of NP coordination ('John and Mary went to market') and the marking of comitative phrases ('John went to market with Mary')? (1: present, 0: absent)

28. Is there an inclusive/exclusive distinction? (1: present, 0: absent)

29. Is there a minimal-augmented system? *i.e. four basic pronominal forms for 1sg, 2sg, 3sg and 1+2, which each can be affixed for plural (or dual etc.)* (1: present, 0: absent)

30. Is there a gender distinction in 3rd person pronouns (or demonstratives, if no 3rd person pronouns)? *either two- or threefold* (1: present, 0: absent)

31. Is there a dual (or unit augmented) in addition to a plural (or augmented) number category in pronouns? (1: present, 0: absent)

32. Are 1st and 2nd persons conflated in any context? (1: present, 0: absent)

33. Are 2nd and 3rd persons conflated in non-singular numbers? *morphologically in any paradigm. Disregard pragmatics/politeness* (1: present, 0: absent)

34. Are person categories neutralized under some conditions? *e.g. in non-singular, under NEG, in certain TAM* (1: present, 0: absent)

35. Is there an opposition between three or more distance terms in the demonstrative system? (1: present, 0: absent)

36. Is elevation morphologically marked in demonstratives? (1: present, 0: absent)

37. Is the opposition visible-non-visible marked on demonstratives? (1: present, 0: absent)

38. Are demonstratives classified? (1: present, 0: absent)

39. Are there declensions (partly) determined by number of the noun? *By noun declensions is meant e.g nouns divided into groups which have formally different sets of morphological marking. Do not include place names which can act as bare adjuncts* (1: present, 0: absent)

40. Are there declensions (partly) determined by gender of the noun? *By noun*

declensions is meant e.g nouns divided into groups which have formally different sets of morphological marking. Do not include place names which can act as bare adjuncts (1: present, 0: absent)

41. Are there nouns which are suppletive for number? *Only answer yes if present for more than 2 (basic) kin terms* (1: present, 0: absent)

42. Can singular number be marked on the noun itself? *Number marking on noun does not count phrase level clitic or reduplication; absence of plural marking does not count as singular marking; exclude derivational forms (e.g. deverbal, deadjectival)* (1: present, 0: absent)

43. Can dual number be marked on the noun itself? *number-marking on N does not count phrase-level clitic or reduplication* (1: present, 0: absent)

44. Can plural number be marked on the noun itself? *number-marking on N does not count phrase-level clitic or reduplication* (1: present, 0: absent)

45. Is number marking prohibited on certain (types of) nouns? *(do not include proper nouns, e.g. place names or personal names)* (1: present, 0: absent)

46. Are there associative plurals? *e.g. Mary-PL = Mary and her family* (1: present, 0: absent)

47. Is there a productive morphologically marked Action/state nominalization (arrive-arrival)? *if a language is precategorial, include the morphological mechanisms to produce such 'nominalizations'* (1: present, 0: absent)

48. Is there a productive morphologically marked Agentive nominalization (sing-er)? (1: present, 0: absent)

49. Is there a productive morphologically marked Object nominalization (sing; song)? (1: present, 0: absent)

50. Are there noun classes/genders? *By noun classes/genders is meant a system of dividing all or almost all of the nouns of a language into morphological classes which determine agreement phenomena beyond the noun itself.* (1: present, 0: absent)

51. Is sex a relevant category in the noun class/gender system? (1: present, 0: absent)

52. Is shape a relevant category in the noun class/gender system? (1: present, 0: absent)

53. Is animacy (without reference to sex) a relevant category in the noun class/gender system? (1: present, 0: absent)

54. Is plant status a relevant category in the noun class/gender system? (1: present, 0: absent)

55. Does the language only have a gender distinction in 3rd person pronouns? (1: present, 0: absent)

56. Is there concord within the NP, i.e. agreement of elements within the NP with the noun class of a noun? *related to class/gender* (1: present, 0: absent)

57. Are there numeral classifiers? *i.e. free or bound morphemes which are non-agreeing, noun categorisation devices, the choice of which is determined by lexical selection* (1: present, 0: absent)

58. Are there possessive classifiers? *i.e. free or bound morphemes which are non-agreeing, noun categorisation devices, the choice of which is determined by lexical selection* (1: present, 0: absent)

59. Is alienable/inalienable a relevant distinction? (1: present, 0: absent)

60. Are there different possessive constructions? (1: present, 0: absent)

61. Can possession be marked by a prefix? *even if only on a restricted numer of kin terms. Emphasis is on *can** (1: present, 0: absent)

62. Can possession be marked by a suffix? *even if only on a restricted numer of kin terms. Emphasis is on *can** (1: present, 0: absent)

63. Can possession be marked on the nominal possessor? (1: present, 0: absent)

64. Can possession be marked on the nominal possessee? (1: present, 0: absent)

65. What is the relative position of possessor and possessed in the attributive possessive construction? (*multistate* 1:Possessor-Possessed; 2:Possessed-Possessor; 3: both)

66. Are there different orders of elements in a possessive phrase for different classes of possession? *emphasis on *for different types of possession** (1: present, 0: absent)

67. What is the counting system? (*multistate* 1:Decimal; 2:Quinary; 3: Body-part tallying; 4: minimal)

68. Do core adjectives (defined semantically as property concepts; value, shape, age, dimension) act like verbs in predicative position? (1: present, 0: absent)

69. Do core adjectives (defined semantically as property concepts; value, shape, age, dimension) used attributively require the same morphological treatment as verbs? (1: present, 0: absent)

70. Is there case marking for core nominal NPs (i.e., S, A or O function)? *for case marking, include any affixal marking which appears in the NP and shows the function of the NP in the clause; do not count adpositions* (1: present, 0: absent)

71. Is there case marking for core pronouns? (1: present, 0: absent)

72. Is there case marking for oblique nominal NPs ? *e.g. locationals, instrumentals, etc. do not count adpositions* (1: present, 0: absent)

73. Is there case marking for oblique pronouns? (1: present, 0: absent)

74. Are there prepositions? (1: present, 0: absent)

75. Are there postpositions? (1: present, 0: absent)

76. Are there adpositions to mark core NPs? (1: present, 0: absent)

77. Are there adpositions to mark oblique NPs? (1: present, 0: absent)

78. Is there a distinction between locational and directional adpositions? (1: present, 0: absent)

79. Do verbs have prefixes/proclitics, other than those that ONLY mark A, S or O (do include portmanteau: A & S + TAM)? *A, S, and O affixes are dealt with in 3.3* (1: present, 0: absent)

80. Do verbs have suffixes/enclitics, other than those that ONLY mark A, S or O (do include portmanteau: A & S + TAM)? (1: present, 0: absent)

81. Can infixation be used on verbs for derivational, aspectual, or voice-changing purposes? (1: present, 0: absent)

82. Is there present tense regularly morphologically marked on the verb? (1: present, 0: absent)

83. Is there past tense regularly morphologically marked on the verb? (1: present, 0: absent)

84. Is there future tense regularly morphologically marked on the verb? (1: present, 0: absent)

85. Are there multiple past or future tenses, distinguishing distance from Time of Reference, marked on the verb? (1: present, 0: absent)

86. Is a distinction between punctual/continuous aspect available as a morphological choice? (1: present, 0: absent)

87. Is a distinction between realis/irrealis mood available as a morphological choice? (1: present, 0: absent)

88. Is there an apprehensive modal category marked on the verb *also known as 'evitative', 'lest', etc* (1: present, 0: absent)

89. Is the S participant (at least sometimes) marked by a suffix/enclitic? *pertains to verb morphology* (1: present, 0: absent)

90. Is the S participant (at least sometimes) marked by a prefix/proclitic? *pertains to verb morphology* (1: present, 0: absent)

91. Is the A participant (at least sometimes) marked by a suffix/enclitic? *pertains to verb morphology* (1: present, 0: absent)

92. Is the A participant (at least sometimes) marked by a prefix/proclitic? *pertains to verb morphology* (1: present, 0: absent)

93. Is the O participant (at least sometimes) marked by a suffix/enclitic? *pertains to verb morphology* (1: present, 0: absent)

94. Is the O participant (at least sometimes) marked by a prefix/proclitic? *pertains to verb morphology* (1: present, 0: absent)

95. Are variations in marking strategies of core participants based on TAM distinctions? *this question refers to variations (if they occur) in 89-94* (1: present, 0: absent)

96. Are variations in marking strategies based on verb classes? *this question refers to variations (if they occur) in 89-94* (1: present, 0: absent)

97. Are variations in marking strategies based on clause type, e.g. main vs subordinate? *this question refers to variations (if they occur) in 89-94* (1: present, 0: absent)

98. Are variations in marking strategies based on person distinctions? *this question refers to variations (if they occur) in 89-94* (1: present, 0: absent)

99. Do verb stems alter according to the person of a core participant? (1: present, 0: absent)

100. Is number ever marked separately from person on the verb? (1: present, 0: absent)

101. Are person, number and any TAM category (i.e. 3 or more categories in all) marked by portmanteau morphemes on verbs? (1: present, 0: absent)

102. Are categories such as person, number, gender related to a single participant discontinuously marked on a verb? (1: present, 0: absent)

103. Are benefactive nominals marked on the verb? (1: present, 0: absent)

104. Can instruments be marked on the verb? (1: present, 0: absent)

105. Can recipients be treated as a transitive object, i.e. as Direct Object? (1: present, 0: absent)

106. Are there syntactically ditransitive verbs? (1: present, 0: absent)

107. Is negation marked morphologically on the verbs? *i.e. affixation, stem alternation, neutralization of some inflection* (1: present, 0: absent)

108. Can locative or direction be morphologically marked on the verb? *Locative as Direct Object ('she sleeps mat') does not qualify* (1: present, 0: absent)

109. Are there suppletive verbs for number of participants? *(list them all if feasible, otherwise give an estimate of the number and/or proportion of nouns)* (1: present, 0: absent)

110. Are there suppletive verbs for tense or aspect? (1: present, 0: absent)

111. Are there conjugation classes? (1: present, 0: absent)

112. Are there (several) verbs which can be used either transitively or intransitively with no morphological marking? *say no if it's only one or two stems; Intended here is the 'break' and 'open' type; not John eats/ eats the bread* (1: present, 0: absent)

113. Is there transitivising morphology (include clitics)? (1: present, 0: absent)

114. Is there morphology (include clitics) to mark a reflexive action? *free word/particle does not count; neither a default P/N co-reference* (1: present, 0: absent)

115. Is there morphology (include clitics) to mark a reciprocal action? *free word/particle does not count; neither a default P/N co-reference* (1: present, 0: absent)

116. Do verbs classify the shape, size, consistency or position of absolutive arguments by means of incorporated nouns, verbal affixes or suppletive verb stems? *not included here are positional verbs that classify a referent in such terms - covered by 127 (*1: present, 0: absent)

117. Is there a copula for predicate nouns? *e.g. John is a teacher* (1: present, 0: absent)

118. Are there serial verb constructions? *(i.e. two or more verbs in juxtaposition, functioning as a single predicate, with no morphology to mark their relationship with each other. Each of the verbs is a separate phonological word but the construction as a whole is expressed in one intonational unit. Morphology is shared to a greater or lesser extent.)* (1: present, 0: absent)

119. Are there modal auxiliaries? (1: present, 0: absent)

120. Are there aspectual auxiliaries? (1: present, 0: absent)

121. Are there tense auxiliaries? (1: present, 0: absent)

122. Is verb compounding a regular process? *(i.e. two or more verb stems acting as one phonological and grammatical word)* (1: present, 0: absent)

123. Are there verb-adjunct (aka light-verb) constructions? *(i.e. constructions involving a non-predicating element expressing the lexical meaning of the construction, in conjunction with a semantically fairly empty verb, which enables the element to function as a predicate by providing the necessary morphology, e.g. eye do for 'see'; or sneeze hit for 'sneeze')* (1: present, 0: absent)

124. Is there incorporation of nouns into verbs a productive intranisitivizing process? (1: present, 0: absent)

125. Is there productive incorporation of other elements (adjectives, locatives, etc.) into verbs? (1: present, 0: absent)

126. Is there one or more existential verb? *exclude e.g. positional verbs (3.8.02)* (1: present, 0: absent)

127. Are there positional (classificatory) verbs? *(i.e. in answer to a question 'Where is the X', does the verb used in the answer depend on the type of referent (e.g. do you have to say 'The X sits/stands/lies/etc on the table'). List them all.* (1: present, 0: absent)

128. Is the verb 'give' morphologically peculiar (different from most other verbs)? *e.g. stem suppletion, different affixation* (1: present, 0: absent)

129. Is there a notably small number, i.e. about 100 or less, of verbs in the language? (1: present, 0: absent)

130. What is the pragmatically unmarked order of S and V in instransitive clauses? (*multistate* 1: SV; 2: VS; 3: both)

131. Is a pragmatically unmarked constituent order verb-initial for transitive clauses? (1:

present, 0: absent)

132. Is a pragmatically unmarked constituent order verb-medial for transitive clauses? (1: present, 0: absent)

133. Is a pragmatically unmarked constituent order verb-final for transitive clauses? (1: present, 0: absent)

134. Is the order of constituents the same in main and subordinate clauses? (1: present, 0: absent)

135. Do clausal objects occur in the same position as nominal objects? (1: present, 0: absent)

136. Is constituent order fixed? *Do not consider 'left or right-dislocation', accompanied by intonational signals* (1: present, 0: absent)

137. Can negation be marked clause-finally? *This includes suffixes on verb-final clauses; prefixes on clause-final verbs do not count; Don't include elliptical 'Pete didn't'* (1: present, 0: absent)

138. Can negation be marked clause-initially? *Don't include elliptical 'Not Mary'* (1: present, 0: absent)

139. Is there a difference between imperative and declarative negation? (1: present, 0: absent)

140. Are verbal and non-verbal predicates marked by the same negator? (1: present, 0: absent)

141. Are S and O conflated morphologically in at least some basic constructions, i.e. simple main clauses? (1: present, 0: absent)

142. Are S and A conflated morphologically in at least some basic constructions, i.e. simple main clauses? (1: present, 0: absent)

143. Are S and O conflated morphologically across clause boundaries, i.e. acting as syntactic pivot? (1: present, 0: absent)

144. Are S and A conflated morphologically across clause boundaries, i.e. acting as syntactic pivot? (1: present, 0: absent)

145. Do S and O operate in the same way, and differently from A, for the purpose of any syntactic construction? (1: present, 0: absent)

146. Is there a morpho-syntactic distinction between predicates expressing controlled versus uncontrolled events or states? (1: present, 0: absent)

147. Is there a morphologically marked passive construction? *morphological marking includes some verbal affixation or some periphrastic element in the VP or clause* (1: present, 0: absent)

148. Is there a morphologically marked antipassive? *morphological marking includes some verbal affixation or some periphrastic element in the VP or clause* (1: present, 0:

absent)

149. Is there a morphologically marked inverse? *i.e. different marking by verbal affixation or pronominal clitics referring to A and O, depending on person, animacy or definiteness* (1: present, 0: absent)

150. Is there clause chaining? *i.e. chains of morphologically stripped-down medial clauses which are dependent on a single clause (usually, but not necessarily, final) for their TAM or participant marking specification* (1: present, 0: absent)

151. Is there a morphologically-marked switch reference system? (1: present, 0: absent)

152. Is there a morphologically marked distinction between simultaneous and sequential clauses? (1: present, 0: absent)

153. Is the verb 'say' or a quotative construction used in desiderative constructions? *(e.g. 'I said for him to go' for 'I wanted him to go')* (1: present, 0: absent)

154. Are causatives formed by serial verb constructions? (1: present, 0: absent)

155. Are causatives formed by bound affixes/clitics? (1: present, 0: absent)

156. Are causatives formed by constructions involving 'say'? (1: present, 0: absent)

157. Is there tail-head linkage? *(i.e. a discourse strategy in which the final verb of one sentence is repeated as the first verb of the next sentence)* (1: present, 0: absent)

158. Are verbs reduplicated? (1: present, 0: absent)

159. Are nouns reduplicated? (1: present, 0: absent)

160. Are elements apart from verbs or nouns reduplicated? (1: present, 0: absent)
