# Supplementary material for: Explaining the Linguistic Diversity of Sahul Using Population Models
Source: PLoS Biol. 2009 Nov 17;7(11):e1000241. doi: 10.1371/journal.pbio.1000241 (PMC2770058; doi:10.1371/journal.pbio.1000241)
Supplement: Text S2 — Sources of language data. The 121 languages investigated in this study along with ISO-639-3 language codes. (0.13 MB PDF) [file pbio.1000241.s005.pdf]

Language Sources

Sources of language data

Language names given with ISO-639-3 (“Ethnologue”) language codes.

Abau [aau]

Lock, Arjen. 1999. Abau Grammar sketch. [ms] Ukarumpa: SIL.

Abui [abz]

Kratochvíl, František. 2007. A grammar of Abui. PhD thesis, Leiden University. Utrecht: LOT.

Aiwoo [nfl]

Næss, Åshild, Fieldnotes.

Ross, Malcolm and Næss, Åshild. 2007. An Oceanic origin for Äiwoo, the language of the Reef Islands? Oceanic Linguistics 46,2:456-498.

Alamblak [amp]

Bruce, Les. 1984. The Alamblak language of Papua New Guinea (East Sepik). Canberra: Pacific Linguistics

Bruce, Les. 1974. Alamblak kinsmen. Kinship studies in Papua New Guinea, ed. by R. Daniel Shaw, 169-186. Ukarumpa: SIL.

Ambulas [abt]

Wilson, Patricia R. 1980. Ambulas Grammar. Workpapers in Papua New Guinea languages, Vol.26. Ukarumpa: SIL.

Arammba [stk]

Boevé, Alma and Marco Boevé. 2003. Grammar essentials of the Arammba language. [ms] Ukarumpa: SIL.

Bali-Vitu [bbn]

Ross, Malcolm. 2002. Bali-Vitu. The Oceanic languages, ed. by John Lynch, Malcolm Ross and Terry Crowley, 362-386. Richmond: Curzon Press.

Van den Berg, René and Peter Bachet. 2006. Vitu grammar sketch. [Data Papers on Papua New Guinea Languages, Volume 51] Ukarumpa: SIL.

Bandjalang [bdy]

Crowley, T. 1978. The middle Clarence dialects of Bandjalang. Canberra: Australian Institute of Aboriginal Studies.

Sharpe, M. C. 2005. Grammar and texts of the Yugambeh-Bundjalung dialect chain in Eastern Australia, Languages of the world/materials 370. Munich: Lincom Europa.

Sharpe, M. C. 2007. A revised view of the verbal suffixes of Yugambeh-Bundjalung. Language description, history and development, ed. by J. Siegel, J. Lynch and D. Eades, 53-78. Amsterdam: John Benjamins.

Banoni [bcm]

Lynch, John and Malcolm Ross. 2002. Banoni. The Oceanic languages, ed. by John Lynch, Malcolm Ross and Terry Crowley, 440-455. Richmond: Curzon Press..

Bardi [bcj]

Bowern, Claire L. 2004. Bardi verb morphology in historical perspective. PhD thesis, Department of Linguistics, Harvard University.

Bauzi [bvz]

Briley, David. 1997. Four grammatical marking systems in Bauzi. Papers in Papuan Linguistics No. 2, ed. by Karl Franklin, 1-131. Canberra: Pacific Linguistics

Belait [beg]

Clynes, Adrian. 2005. Belait. The Austronesian languages of Asia and Madagascar, ed. by Alexander Adelaar and Nikolaus P. Himmelmann, 429-455. London: Routledge/Curzon.

Biak [bhw]

Van den Heuvel, Wilco. 2006. Biak, Description of an Austronesian language of Papua. Utrecht: LOT.

Bilua [blb]

Obata, K. 2003. A grammar of Bilua: A Papuan language of the Solomon Islands. Canberra: Pacific Linguistics.

Bine [bon]

Lillian Fleischmann & Sinikka Turpeinen. 1975. Bine Grammar Essentials. [ms] Ukarumpa: SIL.

Lillian Fleischmann & Sinikka Turpeinen. 1977. Bine phonemes. Workpapers in Papua New Guinea Languages, Vol 22:99-144. Ukarumpa: SIL.

Sinikka Saari. 1992. Organized Phonology Data. Ukarumpa: SIL.

Bininj Gun-wok [gup]

Evans, Nicholas. 2003. Bininj Gun-wok: A pan-dialectal grammar of Mayali, Kunwinjku and Kune. Canberra: Pacific Linguistics.

Bukiyip [ape]

Conrad, Robert J. with Kepas Wogiga. 1991. An outline of Bukiyip grammar. Canberra: Pacific Linguistics.

Buma [tkw]

Tryon, Darrell. 2002. Buma. The Oceanic languages, ed. by John Lynch, Malcolm Ross and Terry Crowley, 573-586. Richmond: Curzon Press.

Burarra [bvr]

Green, R. 1987. A sketch grammar of Burarra. honours thesis, Linguistics, Australian National University.

Glasgow, K. 1981. Burarra phonemes. Australian phonologies: collected papers. Work papers of SIL-AAB, Series A, Volume 5, ed. by B. Waters, 63-90. Darwin: Summer Institute of Linguistics.

Glasgow, K. and Garner, M. 1980. Clause-level tagmemes of Burarra. Papers in Australian linguistics No. 12, ed. by C. Street, H. Geytenbeek, K. Glasgow and M. Garner, 37-82. Canberra: Pacific Linguistics.

Green, R. 2003. Proto Maningrida within Proto Arnhem: evidence from verbal inflectional suffixes.

The non-Pama-Nyungan languages of Northern Australia: comparative studies of the continent's most linguistically complex region, Studies in language change, ed. by N. Evans, 369-421. Canberra: Pacific Linguistics.

Cèmuhî [cam]

Lynch, John. 2002. Cèmuhî. The Oceanic languages, ed. by John Lynch, Malcolm Ross and Terry Crowley, 753-764. Richmond: Curzon Press.

Djambarrpuyngu [djr]

Wilkinson, M. 1991. Djambarrpuyngu a Yolngu variety of Northern Australia. PhD thesis, Linguistics department, Sydney University.

Duke of York [rai]

Davies, Robyn and Fritzell, Lisbeth. 1992. Duke of York Grammar Essentials (Ramoaaina). [ms] Ukarumpa: SIL

Fijian [fij]

Dixon, R.M.W. 1988. A Grammar of Boumaa Fijian. Chicago: University of Chicago Press.

Gapapaiwa [pwg]

McGuckin, Catherine. 2002. Gapapaiwa. The Oceanic languages, ed. by John Lynch, Malcolm Ross and Terry Crowley, 297-321. Richmond: Curzon Press.

Garrwa [gbc]

Belfrage, H. 1992. Aspects of verb and pronoun morphology, semantics and syntax in Garrwa. honours thesis, Linguistics department, University of Melbourne.

Breen, G. 2003. Wanyi and Garrwa comparative data. The non-Pama-Nyungan languages of Northern Australia: comparative studies of the continent's most linguistically complex region, Studies in language change, ed. by N. Evans, 425-62. Canberra: Pacific Linguistics..

Furby, C. E. 1974. Garawa phonology. Papers in Australian linguistics no.7, ed.by Furby, C.E., Hercus, L.A. and Kilham C., 1-16. Canberra: Pacific Linguistics.

Furby, E. S. and Furby, C. E. 1977. A preliminary analysis of Garawa phrases and clauses. Canberra: Pacific Linguistics.

Mushin, I. 2006. Motivations for second position: evidence from North-Central Australia. Linguistic typology 10:287-326.

Mushin, I. 2005. Word order pragmatics and narrative functions in Garrwa. Australian Journal of Linguistics 25:253-73.

Mushin, Ilana. 2005 Narrative functions of clause linkage in Garrwa: a perspective analysis, Studies in Language 29:1, 1-33.

Gizrra [tof]

Van Bodegraven, Nico and Elly. 2004. Gizrra Grammar Essentials. [ms] Ukarumpa: SIL.

Van Bodegraven, Nico and Elly. 2004. Phonology essentials of the Gizrra language. [ms] Ukarumpa: SIL..

Gooniyandi [gni]

McGregor, William B. 1990. A functional grammar of Gooniyandi. Amsterdam: John Benjamins.

Halia [hla]

Allen, Jerry. 1987. Halia Grammar. [Datapapers on Papua New Guinea languages Vol.32] Ukarumpa: SIL.

Hatam [had]

Reesink, Ger. 1999. A grammar of Hatam. Canberra: Pacific Linguistics

Hua [ygr]

Haiman, John. 1980. Hua: A Papuan language of the Eastern Highlands of New Guinea. Amsterdam, John Benjamins.

Iaai [iai]

Lynch, John. 2002. Iaai. The Oceanic languages, ed. by John Lynch, Malcolm Ross and Terry Crowley, 776-791. Richmond: Curzon Press.

Ilocano [ilo]

Vanoverbergh, Morice. 1955. Iloko Grammar. Catholic School Press

Vanoverbergh, Morice. 1968. English-Iloko Thesaurus. Corimacon Inc. Catholic School Press.

Laconsay, Gregorio. 1977. Iluko-English-Tagalog Dictionary

Rubino 2000. Ilocano Dictionary and Grammar

Rubino, Carl. 2005. The Austronesian languages of Asia and Madagascar, ed. by Alexander Adelaar and Nikolaus P. Himmelmann, 326-396. London: Routledge/Curzon.

Imonda [imn]

Seiler, Walter. 1985. Imonda, a Papuan language. Canberra: Pacific Linguistics.

Inanwatan [szp]

De Vries, Lourens. 2004. A short grammar of Inanwatan. Canberra: Pacific Linguistcs.

Isaka [ksi]

Donohue, Mark and Lila San Roque. 2004. I'saka, a sketch grammar of a language of North-Central New Guinea. Canberra: Pacific Linguistics.

Jabêm [jae]

Ross, Malcolm. 2002. Jabêm. The Oceanic languages, ed. by John Lynch, Malcolm Ross and Terry Crowley, 270-296. Richmond: Curzon Press.

Bradshaw, Joel and Francisc Czobor. 2005. Otto Dempwolff's Grammar of the Jabêm language in New Guinea. Honolulu: University of Hawai'i Press.

Kala Lagaw Ya [mwp]

Comrie, B. 1981. Ergativity and grammatical relations in Kalaw Lagaw Ya (Saibai dialect). Australian Journal of Linguistics 1:1-42.

Kennedy, R. J. 1981. Phonology of Kala Lagaw Ya in Saibai dialect. Australian phonologies: collected papers. Work papers of the Summer Institute of Linguistics, Australian Aborigines Branch, series A volume 5, ed. by B. E. Waters, 103-137. Darwin: Summer Institute of Linguistics.

Kennedy, R. J. 1985. Clauses in Kala Lagaw Ya. Aboriginal and Islander grammars: collected

papers, ed. by S. K. Ray, 59-79. Darwin: Summer Institute of Linguistics.

Kennedy, R. J. 1985. Kalaw Kawaw verbs. Aboriginal and Islander grammars: collected papers, ed. by S. K. Ray, 81-103. Darwin: Summer Institute of Linguistics.

Kennedy, R. J. 1985. Kalaw Kawaw verbs: speaker perspective and tense, mood, and aspect. Aboriginal and Islander grammars: collected papers, ed. by S. K. Ray, 105-118. Darwin: Summer Institute of Linguistics.

Ford, K. and Ober, D. 1991. A sketch of Kalaw Kawaw Ya. Language in Australia, ed. by S. Romaine, 118-142. Cambridge: Cambridge University Press.

Bani, E. 1987. Garka a ipika: masculine and feminine grammatical gender in Kala Lagaw Ya. Australian Journal of Linguistics 7:189-201.

Kamasau [kms]

Sanders, Arden and Sanders, Joy. 1994. Kamasau (Wand Tuan) Grammar - Morpheme to sentence. [ms] Ukarumpa: SIL.

Sanders, Arden & Sanders, Joy. 1980. Phonology of the Kamasau language. Papers in New Guinea Linguistics, No.20:111-135. Canberra: Pacific linguistics

Kamoro [kgq]

Drabbe, P. 1953. Spraakkunst van de Kamoro-taal. DenHaag: Martinus Nijhoff.

Kayardild [gyd]

Evans, Nicholas. 1995. A grammar of Kayardild. Berlin: Mouton de Gruyter.

Kele [sbc]

Ross, Malcolm. 2002. Kele. The Oceanic languages, ed. by John Lynch, Malcolm Ross and Terry Crowley, 123-147. Richmond: Curzon Press.

Kewa [kew]

Franklin, K.J. 1971. A grammar of Kewa, New Guinea. Canberra: Pacific Linguistics.

Franklin, K. and Franklin, J. 1978. A Kewa dictionary. Canberra: Pacific Linguistics.

Franklin, K. J. 1981. Existential and pro-verbs in Kewa. Syntax and Semantics in Papua New Guinea languages, ed. by Karl Franklin, 151-171. Ukarumpa: SIL.

Yarapea, A. 2001. On interclausal reference in Kewa. The boy from Bundaberg, ed. by Andrew Pawley, Malcolm Ross and Darrell Tryon, 399-417. Canberra: Pacific Linguistics.

Kilivila [kij]

Lawton, R. 1993. Topics in the description of Kiriwina. Canberra, Pacific Linguistics.

Senft, Gunter. 1986. Kilivila: The language of the Trobriand islanders. Berlin: Mouton de Gruyter.

Kimaragang [kqr]

Kroeger, Paul. 2005. Kimaragang. The Austronesian languages of Asia and Madagascar, ed. by Alexander Adelaar and Nikolaus P. Himmelmann, 397-428. London: Routledge/Curzon.

Kiwai Southern [kjd]

Ray, S. H. 1931. A grammar of the Kiwai language, Fly Delta, Papua, with a Kiwai Vocabulary, by Rev. E. Baxter Riley. Port Moresby: Edward George Baker, Government Printer.

Klon [kyo]

Baird, Louise. Forthcoming. A grammar of Klon : a non-Austronesian language of Alor, Indonesia. Canberra: Pacific Linguistics..

Kobon [kpw]

Davies, John. 1989. Kobon. Croom Helms Descriptive Grammars. London, Routledge.

Koiari [kbk]

Dutton, Tom. 2003. A dictionary of Koiari, Papua New Guinea, with grammar notes. Canberra: Pacific Linguistics

Dutton, Tom. 1993. Possession in Koiari. Language and Linguistics in Melanesia 24.1:39-62.

Kokota [kkk]

Palmer, Bill. 1999. A grammar of the Kokota language, Santa Isabel, Solomon Islands. Department of Linguistics. Sydney, University of Sydney.

Palmer, Bill. 2002. Kokota. The Oceanic languages, ed. by John Lynch, Malcolm Ross and Terry Crowley, 498-524. Richmond: Curzon Press.

Kol [kol]

Reesink, Ger 2002. Fieldnotes

Lindrud, Stellan. 1989. Aspect in Kol. [14-page ms]

Lindrud, S. 2002. Kol noun classes. [26-page ms]

Lindrud, S. 2002. Kol morhpo-syntax. [20-page ms]

Lindrud, S. (nd). Non-verbal predication in Kol. [36-page ms]

Korafe [kpr]

Farr, Cynthia J. M. 1999. The interface between syntax and discourse in Korafe, a Papuan language of Papua New Guinea. Canberra: Pacific Linguistics

Korowai [khe]

Van Enk, Gerrit J. and Lourens de Vries. 1997. The Korowai of Irian Jaya. Oxford: Oxford University Press.

Kove-Kaliai [kvc]

Goulden, Rick. 1996. The Maleu and Bariai languages of West New Britain. Studies in languages of New Britain and New Ireland, ed. by Malcolm Ross, 63-144. Canberra:Pacific Linguistics..

Counts, David R. 1969. A grammar of Kaliai-Kove. [Oceanic Linguistics Special Publication 6.] Honolulu: University of Hawai'i Press.

Kuot [kto]

Lindström, Eva. Fieldnotes.

Lindström, Eva. 2002. Topics in the grammar of Kuot, a non-Austronesian language of New Ireland, Papua New Guinea. PhD thesis, Stockholm University.

Kuuk Thayorre [thd]

Gaby, Alice R. 2006. A Grammar of Kuuk Thaayorre. PhD thesis, Department of Linguistics and Applied Linguistics, University of Melbourne.

Lavukaleve [lvk]

Terrill, Angela. Fieldnotes

Terrill, Angela. 2003. A grammar of Lavukaleve. Berlin: Mouton de Gruyter.

Longgu [lgu]

Hill, Deborah. 2002. Longgu. The Oceanic languages, ed. by John Lynch, Malcolm Ross and Terry Crowley, 538-561. Richmond: Curzon Press

Madak [mmx]

Lee, Robert. n.d. The Madak noun phrase. [ms] Ukarumpa: SIL.

Lee, Robert. 1978. Madak grammar essentials. [ms] Ukarumpa: SIL.

Lee, Robert. 1989. The Madak verb phrase. Language and Linguistics in Melanesia 20, (1–2): 65–114.

Lee, Robert, and Carolyn Lee. n.d.a. Madak discourse. [ms] Ukarumpa: SIL.

Lee, Robert, and Carolyn Lee. n.d.b. The Madak verb phrase. [ms] Ukarumpa: SIL.

Lee, Robert, and Carolyn Lee. n.d.c. Morphophonemics. [ms] Ukarumpa: SIL.

Lee, Robert, and Carolyn Lee. 1976. A tentative phonemic statement of Madak. [ms] Ukarumpa: SIL.

Mairasi [zrs]

Peckham, Lloyd. 2000. Tentative Mairasi grammar. [ms] SIL, Indonesia

Peckham, L. 1987. Mairasi phonology. Workpapers in Indonesian Languages and Cultures Vol. 10:113-146. UNCEN-SIL.

Mali [gcc]

Stebbins, Tonya N. Forthcoming. Mali (Baining) Grammar. Canberra: Pacific Linguistics

Manam [mva]

Lichtenberk, Frantisek. 1983. A grammar of Manam. [Oceanic Linguistics special publications, no 18.] Honolulu: University of Hawai'i Press.

Mangseng [mbh]

Milligan, Lloyd. 1992. Mangseng Grammar Sketch. [ms] Ukarumpa: SIL

Marind [mrz]

Drabbe, P. 1955. Spraakkunst van het Marind. Studia Instituti Anthropos Vol. II. Wien-Mödling: Missiehuis St. Gabriel

Van Baal, J. 1966. Dema, Description and analysis of Marind-anim culture. The Hague: Martinus Nijhoff.

Marquesan, North [mrq]

Lynch, John. 2002. Marquesan. The Oceanic languages, ed. by John Lynch, Malcolm Ross and Terry Crowley, 865-876. Richmond: Curzon Press.

Mutu, Margaret. 2002. Ua Pou: Aspects of a Marquesan Dialect. Canberra: Pacific Linguistics.

Mawng [mph]

Singer, Ruth. 2006. Agreement in Mawng: productive and lexicalised uses of verbal gender agreement in an Australian language. PhD thesis, School of Languages and Linguistics, University of Melbourne (http://eprints.infodiv.unimelb.edu.au/archive/00003242/).

Singer, Ruth. Forthcoming. Untangling strategies for encoding information structure in Mawng. The expression of information structure: a cross-linguistic survey in speech production, Interface explorations, ed. by S. Hellmuth, S. Skopoteas and others. Berlin: Mouton de Gruyter.

Mende [sim]

Hoel, Hanna Marie, Tarja Ikaheimonen, and Michiyo Nozawa. 1994. Mende Grammar Essentials. [ms] Ukarumpa: SIL.

Hoel, Hanna Marie, Tarja Ikaheimonen, and Michiyo Nozawa. 1997. Mende language phonology essentials. manuscript, SIL Ukarumpa.

Hoel, Hanna Marie, Tarja Ikaheimonen, and Michiyo Nozawa. 1998. Alterations and Additions to Mende Grammar Essentials. [ms] Ukarumpa: SIL.

Ikäheimonen, Tarja. 1998. The function of the sentence final position in the Mende language. [ms] Ukarumpa: SIL.

Mengen [mee]

Rath, Daniel D. 1993. Mengen Phonology Essentials. DATA Papers on Papua New Guinea Languages, Vol.40:71-98. Ukarumpa: SIL.

Rath, Daniel. 1986. Mengen Grammar Essentials. [ms] Ukarumpa: SIL.

Madden, Fred. 2000. Logical Relations in Mengen. [ms] Ukarumpa: SIL.

Menggwa Dla [kbv]

De Sousa, Hilario. 2006. The Menggwa Dla language of Papua New Guinea. PhD thesis, University of Sydney.

Menya [mcr]

Whitehead, Carl. 2004. A reference grammar of Menya, an Angan language of Papua New Guinea. PhD thesis, University of Manitoba.

Whitehead, C. 1977. Tense, aspect, mood and modality: verbal morphology in Menya. Papers in Papuan Linguistics 1:245-311. Canberra: Pacific Linguistcs.

Meramera [mxm]

Ohtsuba, Hideki. 1992. Organised Phonology Data for the Melamela language. [ms] Ukarumpa: SIL.

Ohtsuba, Hideki. 1996. Meramera Grammar Essentials. [ms] Ukarumpa: SIL.

Ohtsuba, Hideki. 1999. Meramera interlinearized texts. [ms] Ukarumpa: SIL.

Ohtsuba, Hideki. 2000. Logical relations in Meramera. [ms] Ukarumpa: SIL.

Meriam Mir [ulk]

Piper, N. 1989. A sketch grammar of Meriam Mir. Masters thesis, Australian National University.

Meyah [mej]

Gravelle, Gilles. 2004. Meyah: an east Bird's Head language of Papua, Indonesia. PhD thesis, Vrije Universiteit, Amsterdam.

Motuna [siw]

Onishi, M. 1994. A grammar of Motuna (Bougainville, Papua New Guinea). PhD thesis, Australian National University, Canberra.

Onishi, M. 2002. An annotated word list of the Motuna language. Research Institute for Languages and Cultures of Asia and Africa. Tokyo University of Foreign Studies.

Muna [mnb]

Van den Berg, René. 1989. A grammar of the Muna language. Dordrecht: Foris Publications.

Murrinhpatha [mwf]

Walsh, Michael. 1976. The Murinypata language of north-west Australia. PhD thesis, Australian National University

Walsh, M. 1976. Murinjpata. Grammatical categories in Australian languages, Linguistic series; no. 22., ed. by R. M. W. Dixon, 287-90, 405-08 and 441-44. Canberra: Australian Institute of Aboriginal Studies.

Walsh, M. 1987. The impersonal verb construction in Australian languages. Language Topics. Essays in honour of Michael Halliday, ed. by R. Steele and T. Threadgold, 425-38. Amsterdam: John Benjamins..

Walsh, M. 1996. Body parts in Murrinh-Patha: incorporation, grammar and metaphor. The grammar of inalienability: a typological perspective on body part terms and the part-whole relation, ed. by H. Chappell and W. McGregor, 327-382. Berlin: Mouton de Gruyter.

Walsh, M. 1996. Vouns and nerbs: a category squish in Murrinh-Patha (Northern Australia). Studies in Kimberley languages: in honour of Howard Coate, ed. by W. McGregor, 227-254. Muenchen: Lincom Europa.

Walsh, M. 1997. Noun classes, nominal classification and generics in Murrinhpatha. Nominal classification in Aboriginal Australia, ed. by M. Harvey and N. Reid, 255-292. Amsterdam: John Benjamins.

Blythe, Joe. Forthcoming. From ethical datives to number markers in Murriny Patha. Grammatical change: theory and description, ed. by R. Hendery and J. Hendriks. Canberra: Pacific Linguistics.

Blythe, Joe. Forthcoming. chapter of forthcoming University of Sydney PhD thesis titled ‘Doing referring in Murrinh Patha’.

Street, C. S. 1987. An introduction to the language and culture of the Murrinh-patha. Darwin: Summer Institute of Linguistics, Australian Aborigines Branch.

Street, C. S. 1996. Tense, aspect and mood in Murrinh-Patha. Studies in Kimberley languages: in honour of Howard Coate, ed. by W. McGregor, 205-227. Muenchen: Lincom Europa.

Stanner, W. E. H. 1936. Murinbata kinship and totemism. Oceania 7:186-216.

Mussau [emi]

Ross, Malcolm. 2002. Mussau. The Oceanic languages, ed. by John Lynch, Malcolm Ross and Terry Crowley, 148-166. Richmond: Curzon Press.

Brownie, John and Marjo. 2007. Mussau Grammar Essentials. [Data Papers on Papua New Guinea Languages, Vol. 52.] Ukarumpa: SIL.

Mwotlap [mlv]

Crowley, Terry. 2002. Mwotlap. The Oceanic languages, ed. by John Lynch, Malcolm Ross and Terry Crowley, 597-598. Richmond: Curzon Press.

François, Alexandre. 2004. Chains of freedom: Constraints and creativity in the macro-verb strategies of Mwotlap. Complex Predicates in Oceanic Languages: Studies in the Dynamics of Binding and Boundedness, ed. by Isabelle Bril and Françoise Ozanne-Rivierre,107-143. Berlin: Mouton: Berlin.

François, Alexandre. 2005. A typological overview of Mwotlap, an Oceanic language of Vanuatu. Linguistic Typology 9:115–146.

François, Alexandre. 2006. Serial Verb Constructions in Mwotlap. Serial Verb Constructions: A Cross-Linguistic Typology, ed. by Alexandra Y. Aikhenvald, and R.M.W. Dixon, 223-238. Oxford: OUP.

Nabak [naf]

Fabian, Grace, Edmund Fabian, and Bruce Waters. 1998. Morphology, Syntax and Cohesion in Nabak, Papua New Guinea. Canberra: Pacific Linguistics

Nakanai [nak]

Johnston, R.L. 1980. Nakanai of New Britain, the grammar of an Oceanic language. Canberra: Pacific Linguistics.

Namia [nnm]

Feldpausch, Tom and Becky. 1992. Namia Grammar Essentials. Data papers on Papua New Guinea languages Vol. 39, ed. by John R. Roberts. Ukarumpa: SIL.

Feldpausch, Tom & Becky. 2001. Organised Phonology Data. Ukarumpa: SIL.

Ngarinyin [ung]

Rumsey, Alan. 1982. An intra-sentence grammar of Ungarinjin, north-western Australia. Canberra: Pacific Linguistics.

Coate, H. H. J. and Oates, L. 1970. A grammar of Ngarinjin. Canberra: AIATSIS (Australian Institute of Aboriginal and Torres Strait Islander Studies).

Coate, H. H. J. and Elkin, A. P. 1974. Ngarinjin-English Dictionary. Sydney: Oceania Linguistic Monographs.

Rumsey, A. 1981. Kinship and context among the Ngarinyin. Oceania 51:3, 181.

Scheffler, H.W. 1978. Australian kin classification. Cambridge: CUP.

Scheffler, H.W. 1984. Meaning and use in Ngarinyin Kin classification. Oceania 54,4: 310.

Ngarrinyeri [nay]

Cerin, M. 1994. The pronominal system of Yaraldi. Honours thesis, Department of Linguistics, Faculty of Arts, University of Melbourne.

Horgen, Michael. 2004. The languages of the Lower-Murray. MA thesis, La Trobe University, Melbourne.

McDonald, M. 2002. A study of the phonetics and phonology of Yaraldi and associated dialects, Lincom studies in Australian languages. Munich: Lincom Europa.

Radcliffe-Brown, A. 1918. Notes on the social organization of Australian tribes. The Journal of the Royal Anthropological Institute of Great Britain and Ireland, 48:222-53.

Scheffler, H. W. 1978. Australian kin classification. Cambridge: Cambridge University Press.

Yallop, C. 1975. The Narinjari language 1864-1964, Oceanic linguistic monographs No. 17. Sydney: University of Sydney.

Nggem [nbq]

Paul Etherington. 2002. Nggem morphology and syntax. MA thesis, The Northern Territory University, Australia.

Onge [oon]

Dasgupta, D. and Sharma, S.R. 1982. A handbook of Onge language. Calcutta: Anthropological Survey of India

Abbi, Anvita. 2006. Endangered languages of the Andaman islands. Muenchen: Lincom.

Blevins, Juliette. 2007. A long lost sister of proto-Austronesian? Proto-Ongan, mother of Jarawa and Onge of the Andaman islands. Oceanic Linguistics Vol.46,1:154-198.

Orya [ury]

Fields, Philip C. 1991. A phonology of the Orya language. Papers in Papuan linguistics No.1, ed. by Tom Dutton, 29-56. Canberra: Pacific Linguistics

Fields, Philip C. 1997. Pivot and nominalisation in Orya. Papers in Papuan linguistics No.2, ed. by Karl Franklin, 237-269. Canberra: Pacific Linguistics.

Fields, Philip C. 2000. A summary of Orya Grammar. [ms] SIL, Indonesia.

Rennellese [mnv]

Elbert, Samuel. 1988. Echo of a Culture: A Grammar of Rennell and Belona. Oceanic Linguistics Special Publications No. 22

Elbert, Samuel. 1975. Dictionary of the language of Rennell and Bellona. Part 1: Rennellese and Bellonese to English. Copenhagen: The National Museum of Denmark.

Rotokas [roo]

Robinson, Stuart P. 2007. Split intransitivity in the Rotokas language of Bougainville. PhD thesis [version December 2, 2007].

Rotuman [rtm]

Schmidt, Hans. 2002. Rotuman. The Oceanic languages, ed. by John Lynch, Malcolm Ross and Terry Crowley, 815-832. Richmond: Curzon Press

Roviana [rug]

Corston-Oliver, S. 2002. Roviana. The Oceanic languages, ed. by John Lynch, Malcolm Ross and Terry Crowley, 467-497. Richmond: Curzon Press.

Sama [ssb]

Jun, Akamine. Sama. The Austronesian languages of Asia and Madagascar, ed. by Alexander Adelaar and Nikolaus P. Himmelmann, 377-396. London: Routledge/Curzon.

Selepet [spl]

McElhanon, Kenneth A. 1970. Selepet phonology. Canberra: Pacific Linguistics.

McElhanon, Kenneth A. 1972. Selepet grammar, Part I, From Root to Phrase. Canberra: Pacific Linguistics.

Siar [sjr]

Ross, Malcolm. 2002. Siar. The Oceanic languages, ed. by John Lynch, Malcolm Ross and Terry Crowley, 410-425. Richmond: Curzon Press.

Sisiqa [qss]

Ross, Malcolm. 2002. Sisiqa. The Oceanic languages, ed. by John Lynch, Malcolm Ross and Terry Crowley, 456-466. Richmond: Curzon Press.

South Efate [erk]

Thieberger, Nicholas A. 2006. A grammar of South Efate, an Oceanic language of Vanuatu. [Oceanic Linguistics Special Publications no.33] Honolulu: University of Hawai'i Press.

Sudest [tgo]

Anderson, Mike and Malcolm Ross. 2002. Sudest. The Oceanic languages, ed. by John Lynch, Malcolm Ross and Terry Crowley, 322-346. Richmond: Curzon Press.

Sulka [sua]

Reesink, Ger. 2005. Sulka of East New Britain, a mixture of Oceanic and Papuan traits. Oceanic Linguistics 44-1:145-193

Schneider, Jos. 1942. Grammatik der Sulka-Sprache. [Typescript]

Tharp, Doug. 1996. Sulka Grammar Essentials. DATA Papers on Papua New Guinea Languages, Vol. 42:77-179. Ukarumpa: SIL.

Sye [erg]

Crowley, Terry. 1998. An Erromangan (Sye) Grammar. Oceanic Linguistics no. 27.

Crowley, Terry. 2002. Sye. The Oceanic languages, ed. by John Lynch, Malcolm Ross and Terry Crowley, 694-722. Richmond: Curzon Press.

Taba [mky]

Bowden, John. 2001. Taba: Description of a South-Halmahera language. Canberra: Pacific Linguistics.

Tagalog [tgl]

Himmelmann, Nikolaus P. 2005. Tagalog. The Austronesian Languages of Asia and Madagascar, ed. by K. Alexander Adelaar and Nikolaus P. Himmelmann. London: Routledge.

Schachter, Paul. 1983. Tagalog reference grammar. University of California Press.

Takia [tbc]

Ross, Malcolm. 2002. Takia. The Oceanic languages, ed. by John Lynch, Malcolm Ross and Terry Crowley, 216-248. Richmond: Curzon Press.

Tauya [tya]

MacDonald, Lorna. 1990. A grammar of Tauya. Berlin: Mouton de Gruyter.

Telefol [tlf]

Healey, Alan. 1964 [reprinted 1981] Telefol phonology. PL B-3. Canberra: Pacific Linguistics.

Healey, Phyllis M. 1965. Telefol noun phrases. PL B-4:1-51. Canberra: Pacific Linguistics.

Healey, Phyllis M. 1965. Telefol verb phrases. PL A-5:27-53. Canberra: Pacific Linguistics.

Healey, Phyllis M. 1965. Telefol clause structure. PL A-5:1-26. Canberra: Pacific Linguistics.

Healey, Phyllis M. 1966. Levels and chaining in Telefol sentences. PL B-5: 1-64.

Healey, Phyllis M. 1964. Teleéfoól quotative clauses. PL A-3: 27-34 Canberra: Pacific Linguistics.

Healey, Phyllis and Alan. 1977. Telefol dictionary. Canberra: Pacific Linguistics.

Healey, Alan. 1962. Linguistic aspects of Telefomin kinship. Anthropological Linguistics 4(7): 14-28.

Healey, Alan. 1974. A problem of Telefol verb classification. Work Papers in Papua New Guinea languages 7:167-175. Ukarumpa: SIL.

Tidore [tvo]

Van Staden, Miriam. 2000. Tidore, a linguistic description of a language of the North Moluccas. PhD thesis, Leiden University.

Tiwi [tiw]

Lee, Jennifer. 1987. Tiwi today: a study of language change in a contact situation, Pacific Linguistics C-96. Canberra: Pacific Linguistics.

Lee, Jennifer R. 1993. (compiler) Ngawurranungarumagi nginingawila ngapangiraga - Tiwi-English dictionary, Summer Institute of Linguistics, Darwin, Northern Territory, Australia.

Lee, Jennifer R. 2006. Tiwi. Encyclopedia of langauge and linguistics, volume 12, ed. by K. Brown. Boston: Elsevier. 720-23.

Osborne, C. R. 1974. The Tiwi language. Canberra: Australian institute of Aboriginal studies press.

Tobelo [tlb]

Holton, Gary. 2003. Tobelo. Muenchen: Lincom.

Tolai [ksd]

Mosel, Ulrike. 1980. Tolai and Tok Pisin, The influence of the substratum on the development of New Guinea Pidgin. Canberra: Pacific Linguistics.

Mosel, Ulrike. 1982. Local Deixis in Tolai. Here and There, cross-linguistic studies on Deixis and Demonstration, ed. by Jürgen Weissenborn and Wolfgang Klein, 111-132. Amsterdam: John Benjamins.

Mosel, Ulrike. 1984. Tolai syntax and its historical development. Canberra: Pacific Linguistics.

Tsou [tsu]

Zeitoun, Elizabeth. 2005. Tsou. The Austronesian languages of Asia and Madagascar, ed. by Alexander Adelaar and Nikolaus P. Himmelmann, 259-290. London: Routledge/Curzon.

Tungag [lcm]

Stamm, J. 1988. A grammar of the Lavongai language. Lavongai materials, ed. by J. Stamm, T. Donaldson, C. H. Beaumont and M. J. Lloyd, 1-46. Canberra: Pacific Linguistics.

Ulithian [uli]

Lynch, John. 2002. Ulithian. The Oceanic languages, ed. by John Lynch, Malcolm Ross and Terry Crowley, 792-803. Richmond: Curzon Press.

Sohn, Ho-min and Bender, Byron. 1973. A Ulithian Grammar. Canberra: Pacific Linguistics.

Umanakaina [gdn]

Evensen, Sigmund. 1996. Umanakaina grammar, from morpheme to discourse. [ms] Ukarumpa: SIL.

Una [mtg]

Louwerse, Jan. 1988. The morphosyntax of Una in relation to discourse structure. Canberra: Pacific Linguistics.

Uradhi [urf]

Crowley, T. 1983. Uradhi. Handbook of Australian languages, volume 3, ed. by R. M. W. Dixon and B. J. Blake, 306-430. Canberra: Australian National University Press.

Usan [wnu]

Reesink, Ger P. 1987. Structures and their functions in Usan, a Papuan language of Papua New Guinea. Amsterdam: John Benjamins.

Warlpiri [wbp]

Hale, K. 1967. Lessons in Walbiri. Cambridge, Massachusetts: M.I.T.

Hale, K. 1973. Person marking in Warlbiri. A Festschrift for Morris Halle, ed. by S. Anderson and P. Kiparksky, 308-344. New York: Holt, Rinehart and Winston.

Hale, K. 1976. Elementary remarks on Walbiri orthography, phonology and allomorphy. Cambridge, Massachusetts: M.I.T.

Hale, K. 1981. Preliminary remarks on the grammar of part-whole relations in Warlpiri. Studies in Pacific languages and cultures in honour of Bruce Biggs, ed. by J. Hollyman and A. Pawley, 333-344. Auckland: Linguistic Society of New Zealand.

Hale, K. and Bittner, M. 1995. Remarks on definiteness in Warlpiri. Quantification in natural languages, Studies in linguistics and philosophy; v. 54., ed. by E. W. Bach, E. Jelinek, A. Kratzer and B. H. Partee, 81-105. Dordrecht: Kluwer Academic Publishers.

Nash, D. 1980. Topics in Warlpiri grammar. PhD thesis, Linguistics and Philosphy, M.I.T.

Simpson, J. 1991. Warlpiri morpho-syntax. A lexicalist approach, Studies in natural language and linguistic theory 23. Dordrecht: Kluwer.

Simpson, J. Forthcoming. Expressing pragmatic constraints on word order in Warlpiri. Festschrift for Joan Bresnan, ed. by A. Zaenen, J. Simpson, C. Manning and J. Grimshaw. Stanford: CSLI.

Wilkins, D. P. 2000. Ants, ancestors and medicine: a semantic and pragmatical account of classifier constructions in Arrernte (Central Australia). Systems of nominal classification, ed. by G. Senft, 147-216. Cambridge: Cambridge University Press.

Waskia [wsk]

Ross, Malcolm with John Natu Paol. 1978. A Waskia grammar sketch and vocabulary. Canberra: Pacific Linguistics.

Wuvulu-Aua [wuv]

Hafford, James Alton. 1999. Elements of Wuvulu grammar (Papua New Guinea). MA thesis, The University of Texas at Arlington.

Xârâcùù [ane]

Lynch, John. 2002. Xârâcùù. The Oceanic languages, ed. by John Lynch, Malcolm Ross and Terry Crowley, 765-775. Richmond: Curzon Press.

Yagaria [qgr]

Renck, G.L. 1975. A grammar of Yagaria. Canberra: Pacific Linguistics

Yessan-Mayo [yss]

Foreman, Velma. 1974. Grammar of Yessan-Mayo. [Language Data, Asian-Pacific series, number 4] Santa Ana, California: SIL.

Foreman, Velma and Helen Marten. 1973. Yessan-Mayo phonemes. [Workpapers in Papua New Guinea Languages, Volume 2] Ukarumpa: SIL.

Yimas [yee]

Foley, William A. 1991. The Yimas language of New Guinea. Stanford, California: Stanford University Press

Yélî Dnye [yle]

Levinson, Stephen. Fieldnotes

Levinson, Stephen. Forthcoming. A grammar of Yélî Dnye, the Papuan language of Rossel Island.
